# Neural stem cell research in Africa: current realities and future prospects

**DOI:** 10.1242/bio.059574

**Published:** 2022-11-03

**Authors:** Idris A. Azeez, Ifeoluwa O. Awogbindin, Juliet N. Olayinka, Royhaan O. Folarin, Abubakar S. Adamu, Lydia D. Ior, Asmau M. Shehu, Abubakar I. Mukhtar, Olufunke F. Ajeigbe, Aghariagbonse O. Emokpae, Ifukibot L. Usende, Bamidele R. Babatunde, Yusuf Yusha'u, Oladiran I. Olateju, Ronald Kamoga, Ayoola I. O. Benson, Kenneth C. Oparaji, Idowu O. Owemidu, Musa O. Iliyasu, Maryam I. Imam, James O. Olopade

**Affiliations:** ^1^ Department of Veterinary Anatomy, University of Jos, Jos, 930001 Nigeria; ^2^ Department of Biochemistry, University of Ibadan, Ibadan, 200005, Nigeria; ^3^ Department of Pharmacology and Therapeutics, Afe Babalola University, Ado-Ekiti, 360001 Nigeria; ^4^ Department of Anatomy, Olabisi Onabanjo University, Ago-Iwoye, 120107 Nigeria; ^5^ Department of Human Anatomy, Ahmadu Bello University, Zaria, 810107, Nigeria; ^6^ Department of Pharmacology, University of Jos, Jos, 930001, Nigeria; ^7^ Department of Human Anatomy, Federal University Dutse, Dutse, 720223, Nigeria; ^8^ School of Anatomical Sciences, University of the Witwatersrand, Johannesburg, Wits 2050, South Africa; ^9^ Department of Physical and Chemical Sciences, Biochemistry Programme, Elizade University, Ilara-Mokin, 340112, Nigeria; ^10^ Pharmacy Department, University of Benin Teaching Hospital, Benin city, 300001, Nigeria; ^11^ Department of Veterinary Anatomy, University of Abuja, Abuja, 900105, Nigeria; ^12^ Department of Human Physiology, Ahmadu Bello University, Zaria, 810107, Nigeria; ^13^ Department of Pharmacology and Therapeutics, Mbarara University of Science and Technology, Mbarara P.O. Box 1410, Uganda; ^14^ Department of Human Anatomy, Elizade University, Ilara-Mokin, Abakaliki, 482131 Nigeria; ^15^ Department of Physiology, Alex Ekwueme Federal University Ndufu-Alike, Abakaliki, 482131, Nigeria; ^16^ Department of Physiology, Kogi State University, Anyigba, 272102, Nigeria; ^17^ Department of Anatomy, Kogi State University, Anyigba, 272102, Nigeria; ^18^ Department of Veterinary Anatomy, University of Ibadan, Ibadan, 200005, Nigeria

**Keywords:** Neural stem cells, Brain research, Africa, Adult neurogenesis

## Abstract

Neural stem cells (NSCs) are immature progenitor cells that are found in developing and adult brains that have the potential of dividing actively and renewing themselves, with a complex form of gene expression. The generation of new brain cells in adult individuals was initially considered impossible, however, the landmark discovery of human neural stem cells in the hippocampus has been followed by further discoveries in other discreet regions of the brain. Investigation into the current state in Africa of the research and use of NSCs shows relatively limited activities on the continent. Information on the African application of NSCs for modelling disease mechanisms, drug discovery, and therapeutics is still limited. The International Brain Research Organization (IBRO)-African Regional Committee (ARC), with support from the Company of Biologists, and the Movement Disorder Society, sponsored the first African Basic School on NSC in Ibadan, Nigeria, with the vision of bringing together young neuroscientists and physicians across different fields in neuroscience to learn from leaders who have applied NSCs in stem cell research, the pathophysiology of neurodegenerative diseases, neuroanatomy, and neurotherapeutics. Twenty early-career researchers in academic institutions at junior and senior faculty cadres were selected from South Africa, Uganda and Nigeria. The students and organizer of the school, who wrote this review on the state of NSCs research in Africa, recommended the following: (1) other African countries can take a cue from South Africa and Nigeria in probing the phenomena of adult neurogenesis in unique animal species on the continent; (2) Africa should leverage the expertise and facilities of South African scientists and international collaborators in scaling up NSC research into these unique species and (3) Centers of Excellence should be established on the continent to serve as research hubs for training postgraduate students, and facilities for African scientists who trained overseas on NSCs.

## Teaser

**Summary:** A review of stem cell research on the brains of animals in Africa.

## Background

Neural stem cells (NSCs) are immature progenitor cells found in the developing and adult brain with the potential to divide actively, self-renew, showing a complicated form of gene expression that differs in space and time (Ivanova et al., 2002; Ramalho-Santos et al., 2002). It was initially thought that the generation of new brain cells in adult individuals was entirely impossible, owing to the age-long belief that new cells could never fully integrate themselves into the subsisting complex nervous system. However, there was a landmark discovery by Thomson et al., published in 1998, of human neural stem cells in the hippocampus, a region reputable for memory formation and storage, which was followed by further discoveries in other regions such as the olfactory bulbs, striatum, septum and the spinal cord (Thomson et al., 1998).

Neurogenesis occurs in four phases, namely cell proliferation, migration, cell survival and neuronal differentiation. The first reliable scientific proof of how newly generated neurons are formed in the adult rat brain was reported in 1965 (Altman and Das, 1965), after which, Goldman and Nottebohm reported the process of neurogenesis in adult birds (Goldman and Nottebohm, 1983).

In the developing brain (embryonic and fetal stage), the neural stem cells are situated in the ventricular zone (VZ). During the initial developmental stages, the neural tube cavity gives rise to the ventricular system. The neural tube cavity is surrounded by an epithelial monolayer and made up of stem cells (neuroepithelial progenitors, NEPs), which develop into the neuronal and glial cells of the future brain (Grabel, 2012). Meanwhile, the adult NSCs originate mainly from two regions of the adult brain: the dentate gyrus (subgranular zone, SGZ) of the hippocampus and the lateral ventricles subventricular zone (SVZ) (Alvarez-Buylla and Lim, 2004), although some stem cells might also exist in other regions of the brain such as the cerebral cortex, olfactory lobe, as well as in the spinal cord central canal (Rietze et al., 2000; Weiss et al., 1996).

The unique features of the adult mammalian neural stem cell lie in their ability to differentiate, self-renew (being able to form an identical copy of itself), quiescence, and exist in the adult brain. Based on their morphology, molecular marker expression and proliferation kinetics, NSCs in the adult brain can be differentiated. Such differentiation can occur in cells located in the hippocampal dentate gyrus, which are called type 1 radial glial-like neural stem cells (NSCs). These cells are quiescent stem cells with radial processes that cover the whole granule cell layer (Mu et al., 2010; Suh et al., 2009). The characteristic feature of these cells is that they express specific molecular markers like the glial fibrillary acid protein (GFAP), Sox2 and Nestin. These cells differentiate into intermediate progenitor cells (IPCs) – non-radial type 2 cells that express Sox2 and Nestin, but not GFAP – then later form neuroblasts after proliferation (Berg et al., 2015). The generated neuroblasts migrate along the SGZ, and transform into immature neurons that migrate radially into the granular cell layer, where they form the dentate granule neurons (Sun et al., 2015). On the other hand, in the SVZ, there are three types of NSCs known as types A, B and C. The type B cells stretch from the basal process and the apical process before generating amplifying progenitors called C cells. These type C cells then divide to form neuroblasts (A cells) (Mirzadeh et al., 2008).

Certain technologies such as direct extraction from primary tissues (tissue graft), differentiation from pluripotent stem cells and transdifferentiation from somatic cells, have been used to obtain NSCs from different sources (Tang et al., 2017). These sources of NSCs include the human embryonic stem cells (heSC), human fetal brain-derived neural stem/progenitor cells, human induced pluripotent stem cells (hiPSC), mesenchymal stem cells (Buganim et al., 2013; Ling et al., 2009; Satija et al., 2009; Takahashi and Yamanaka, 2006; Thomson et al., 1998). However, through direct reprogramming, NSCs have been obtained from astrocytes (Corti et al., 2012). Fibroblasts that are derived from patients with neurodegenerative diseases have also been a source of induced pluripotent stem (iPS) cells (Bellin et al., 2012). In recent times, new human NSC systems have been derived from iPS cells (Falk et al., 2012; Reinhardt et al., 2013). Of note, each of these types of stem cells possesses some features and benefits. Thus, the reason for using any of these cells depends on what it is being used for and the expected outcomes.

The NSCs have been tools for translational research. They have paved the way for studies on the mechanisms of some nervous disorders (Höing et al., 2012; Huang et al., 2011; Kim et al., 2012; McLaren et al., 2013; Wang et al., 2012), as well as in the modelling (Brennand et al., 2011) and treatment (such as in transplantation, drug screening) of neurological conditions like epilepsy, traumatic brain injury, stroke, cerebral palsy, neonatal hypoxic-ischemic encephalopathy, spinal cord injury (Chau et al., 2014; Chen et al., 2013; Nemati et al., 2014; Salazar et al., 2010; Shetty, 2012; Zhu et al., 2006), in neurodegenerative diseases like Alzheimer's disease, Huntington's disease, Parkinson’s disease (Blurton-Jones et al., 2014; Jones et al., 2016; Nizzardo et al., 2014; Wu et al., 2015; Xu et al., 2006; Yang and Yu, 2009; Zuo et al., 2015), and, also in the modelling of neurotoxicological researches (Gorba and Conti, 2013; Haidet-Phillips et al., 2011; Radio and Mundy, 2008).

## Africa and NSC research capacities: the role of training

**Fig. 1.**
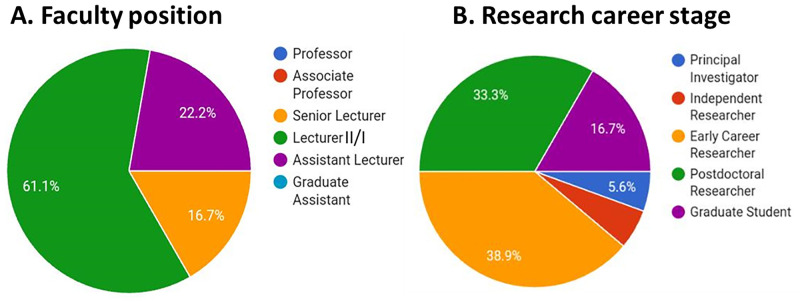
**Pie-chart illustrating the career stages of participants at the 2021 Ibadan NSC School.** (A) Faculty position, (B) research career stage of the trainees at the school. *n*=20.

The current state in Africa with regard to the use of NSCs for research shows that only a handful of research papers on NSCs have been published. Information on the application of NSCs for modelling disease mechanisms and drug discovery, including treatment, is still few and far between. The question of the future prospects of NSC research in Africa needs unraveling. The International Brain Research Organization (IBRO)-African Regional Committee (ARC), with support from the Company of Biologists and the Movement Disorder Society, sponsored the first Basic School on NSC, with the vision of bringing together young neuroscientists and physicians across different fields in neuroscience to learn from leaders who have applied NSC in stem cell research, the pathophysiology of neurodegenerative diseases, neuroanatomy, and neurotherapeutics. The School, themed *Neural Stem Cells: The Biology and Therapeutic Applications* was held at the University of Ibadan from November 30th to December 4th, 2021. Organized by Professor James Olopade, the Ibadan IBRO-ARC Basic School was a giant step toward changing the narratives of NSC in Africa. Twenty early-career researchers in academic institutions at junior and senior faculty cadres were selected from South Africa, Uganda and Nigeria, with 61.1% being lecturers II/I, 22.2% assistant lecturers and 16.7% senior lecturers (Fig. 1A). Of these, there were two doctoral students in their later years and one early principal investigator, while participants of the early-career and postdoctoral cadres constituted the largest proportions (72.2%) of the trainees as illustrated in Fig. 1B. The 4-day session included excellent talks from speakers across three continents on the biology and applications of NSCs. Jeffery Schweitzer of Massachusetts General Hospital, MA, USA, spoke on ‘Stem cell therapy and Parkinson's disease, current and future trends’; Federico Calegari of the Centre for Regenerative Therapies in Dresden, Germany, on ‘Salamanders: model organism of central nervous system regeneration’; Carine Nguemeni of the University Hospital Würzburg, Germany, on ‘Physiology of the cerebellum and its role’; Paul Manger of the University of Witwatersrand, South Africa, on ‘Adult neurogenesis in the mammalian brain’; and Hassan Bassem of the Paris Brain Institute, France, spoke on the ‘Transcriptional control of neuronal development in time and space (flies and mice)’. Together with the organizer, Professor James Olopade, who gave a preparatory talk on ‘International networking on locally sourced rodents: Expanding the frontiers of home front’; other Nigerian speakers included Amos Abolaji, University of Ibadan, who presented, ‘Modelling Parkinson's disease in *drosophila*: promising therapeutic strategies’; and Mathew Olude of the Federal University of Agriculture, who delivered a talk on ‘Stem cells: what is it used for?’. Each presentation provided insights on various applications of NSCs.

At the instance of the Company of Biologists and under the guidance and tutelage of Olopade, the entire body of students of the school were mandated to form a committee to write a review on current realities of NSC research in Africa. In this review, we explored the uptake and application of NSCs and related studies by African researchers. We performed a PubMed search to examine the current realities in Africa concerning research activities on NSCs, and also project the potential prospects of NSC research in Africa.

## Current realities of NSC research in Sub-Saharan Africa

### Neurogenesis studies in animals of African origin dominates

To provide an insight into the NSCs related research activities in Africa, we probed published articles on NSC and neurogenesis using the keywords ‘neural stem cell’, ‘neurogenesis’ and ‘Africa’. Using the three keywords separated with ‘and’, there were 18 hits. We then split the three keywords into two combinations: ‘neurogenesis and Africa’ and ‘neural stem cell and Africa’. The former returned with 70 publications, while the latter showed 54 results. We next pooled the publications together and removed duplicated articles to yield 105 articles. These articles were published between 1993 and 2022, although 93% were from 2013. Of these, 65 articles were removed for being false hits. They either mentioned (but did not investigate) neural stem cells, Africa, and/or neurogenesis in the paper or investigated peripheral stem cells. in addition, some were international papers that investigated viruses of African origin in NSCs.

Of the remaining 45 articles, the majority (80%) were neurogenesis-related, including nine review articles (Ihunwo et al., 2016, 2022; Lipp, 2017; Oosthuizen, 2017; Martínez-Cerdeño et al., 2018; Bruguier et al., 2020; Yang et al., 2020; Dlamini et al., 2021; Samodien and Chellan, 2021). The focus of the remaining neurogenesis papers were similar, with the majority exploring neurogenesis across animal species (Table 1) including mole-rats (Amrein et al., 2014; Oosthuizen and Amrein, 2016), birds (Mazengenya et al., 2020, 2018), African elephants (Patzke et al., 2014b), primates (Fasemore et al., 2018), afrotherian mammals (Patzke et al., 2014a), microchiropterans (Chawana et al., 2014), African giant rats (Olude et al., 2014) and four-striped mice (Olaleye and Ihunwo, 2014). For instance, a study has shown the adult neurogenesis pattern in a giant otter shrew, which appears similar to that in other mammals (Patzke et al., 2013), although, it did show the possible novel presence of neuronal precursor cells in the anterior commissure (Patzke et al., 2013). In another study, adult neurogenesis was observed in eight megachiropteran species, and provided some important observable clues on the neural features in the megachiropterans, which phylogenetically align them to primates (Chawana et al., 2013). Olude and colleagues were able to show the pattern of adult neurogenesis in the African giant rat in 2014. The study revealed a zone of intense proliferating cells within the juvenile and adult brains’ dentate gyrus which could be related to a role in the cognitive activity of landmine detection and diagnosis of tuberculosis. The study further suggested that the African giant rat could be modelled for olfactory training and some experimental research (Olude et al., 2014).

**Table 1.**
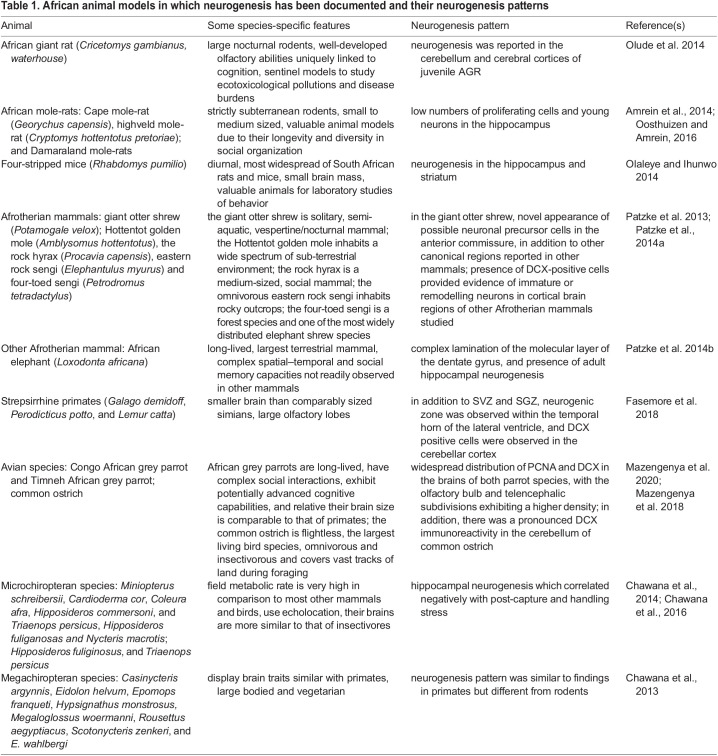
African animal models in which neurogenesis has been documented and their neurogenesis patterns

To a lesser extent, other studies profiled neurogenesis in rodents and other animals following exposure to alcohol (Olateju et al., 2018), irradiation (Zanni et al., 2021), antiretroviral therapy in diabetes (Asouzu Johnson et al., 2022), neonicotinoid pesticide clothianidin (Maeda et al., 2021), cyanobacterial β-N-methylamino-L-alanine (Scott and Downing, 2018), or polyphenolics (Sasaki et al., 2019). These studies explore neurogenesis majorly with *in-situ* immunohistochemical staining of proliferating cells. Interestingly, laboratories in South Africa and Nigeria prominently engaged in probing the phenomena of adult neurogenesis in uncommon animals and rodents in Africa. Of note, we have succinctly summarized the African animal models in which neurogenesis has been documented and the distinct, specific neurogenic niches in the various African animal models, as shown in Table 1.

### A handful of studies on NSCs in Africa

In the last decade, a few studies on NSCs from researchers on the African continent have emerged. Our search captured nine articles including two reviews (George et al., 2019; Van Rensburg et al., 2020). These studies used NSCs to confirm the implications of rare and sporadic familial human disease related to neurogenesis during development (Fan et al., 2014; Law et al., 2014; Chen et al., 2015) or interrogate mechanisms underlying developmental (Fan et al., 2014; Chen et al., 2015; Kannan et al., 2017; FitzPatrick et al., 2018) and adult neurogenesis (Olateju et al., 2021). These studies also generated disease-specific hiPSCs to foster a deeper understanding of such diseases that would aid personalized treatment, and assess targeted therapy (Fan et al., 2014; Law et al., 2014; Chen et al., 2015). Of the seven original research studies, three (Chen et al., 2015; Fan et al., 2014; Law et al., 2014) were translational, describing diseases in certain patients, including the identification of responsible mutations, before studying the outcomes of mutations in patient's neural cells derived from induced pluripotent stem cells. In this category of studies, which connected the phenotypes of genetic diseases to defects in developmental neurogenesis, Law et al. (2014) interrogated the effect of truncating mutations in formin 2 (FMN2) in human cells. The authors identified the mutations by next-generation sequencing of samples from two independent families with autosomal-recessive intellectual disabilities and confirmed that it causes defects in synaptic density in mice. To verify similar defective synaptic density in human cells, the authors used neural cells reprogrammed from patients’ fibroblast-derived iPSCs. They observed a 43% reduction in synaptic density in diseased cells relative to healthy cells. However, these translational studies were led by leading scientists outside of Africa but with affiliations with African institutions.

Another three articles were basic experimental studies. Two of these featured prominently Africa-based scientists (Kannan et al., 2017; Olateju et al., 2021) with the third containing an author affiliated with an African institution (FitzPatrick et al., 2018). The Kannan group followed up on the roles of the less-characterized microtubule-modulating WD40-repeat (WDR) proteins in maintaining neuronal morphology using mice lacking WDR47. They observed that deficiency of WDR47 resulted in severe microcephaly, suggesting a disruptive developmental neurogenic process. They further demonstrated that WDR47 is essential for the proliferation of progenitor cells and neuronal survival during corticogenesis by monitoring WDR47 cortical expression, cortical plate thickness as well as progenitors and neuronal survival across embryonic day 12.5 to postnatal day 2 (Kannan et al., 2017). Similarly, in mice lacking mitogen and stress-activated protein kinase 1 (MSK)-1 activity, Olateju et al., (2021) showed with Ki-67 and doublecortin staining that enrichment-sensitive MSK1 does not influence the proliferation dynamics of neuronal precursors, but regulates the number of differentiated neuronal cells as doublecortin-immunopositive cells increased in the hippocampal subgranular zone (SGZ) following exposure to an enriched environment. In contrast to the studies on neurogenesis described above, these two studies employed genetically modified mice to understand fundamental mechanisms, albeit without extracting NSCs or applying iPSCs. This confirms that basic experimental studies using the NSC approach are not yet established on the continent. On this ground, we included the eighth article by Africa-based scientists because they explored the differentiation of adipose mesenchymal stem cells into neural cells using photobiomodulation (George et al., 2020). The expertise and facility of these authors can be leveraged in scaling up neural stem cell research and training in Africa.

## Prospect for the future of neural stem cell research in Africa

### Dearth of NSC researchers on the continent requires capacity development

According to Simpkin et al. (2019), 15% of the global population is accounted for by Africa, meanwhile this continent is troubled by 25% of the global disease burden. Additionally, the African continent is characterized by the world's largest human genetic diversity, and this poses an important implication for understanding human diseases (Campbell and Tishkoff, 2008), especially neurological dysfunctions and disorders (Little et al., 2017; Akinyemi et al., 2016). There is a huge potential for translating stem cell technology into clinical treatments in Africa, particularly for life-threatening, neurodegenerative and non-communicable diseases which abound in the continent (Gage, 2000). In general, neuroscience research in Africa remains sparse (Maina et al. 2021), and African neuroscience researchers rely on funding (especially from international sources) that are mostly hinged on research questions that are not of direct relevance for Africa (Donald et al., 2022).

Additionally, African scientists in many fields remain disproportionately disconnected from increasingly networked international stem cell research communities. One way to address this challenge is through training and collaboration both locally and internationally. This requires a coalition of stakeholders, both affiliated and resident, to discuss and chart a path for effective uptake of NSC for research, translational and regenerative values. Pivotal in such discussion is the need for more capacity building, which demands that institutions work together to foster research, especially in those areas where equipment is too expensive for a single institution or country to shoulder. Collaborations in NSC research necessitate inclusive partnerships based on common principles, visions and goals, which is key for more sustainable development of capacity, including sharing of pieces of literature, laboratories, knowledge and equipment. Professor Bernard Nthambeleni, the Vice-Chancellor and Principal of the University of Venda, South Africa, said: ‘Collaborations also open up opportunities for postgraduate researchers to move amongst institutions, learning from experts in the field, and being exposed to new contexts’. He noted that collaboration begets more collaboration (Fish, 2021). Conferences and training are ways to establish networks that spur mutual collaborations which will provide complementary expertise, communication between disciplines and the ability to share facilities.

### Impacts of the IBRO-ARC school on NSCs, Ibadan 2021

The school provided opportunities for professional development covering topics on mentoring and afforded good occasions for networking. Importantly, the selection committee provided feedback on abstracts submitted during the call for applications, as well as the presentations rendered by the students touching on the style of presentation, design of slides and the science.

Practical sessions during the school demonstrated the principle of immunohistochemistry, a battery of rodent behavioural tests, bovine brain dissection, and a virtual session from Federico Calegari's laboratory on embryo extraporation technique. Students who attended the school commented that they were able to expand their knowledge on NSC, learn techniques, and were afforded an environment for networking, opportunity to interact with mentors and learn new skill sets, which were adaptable upon return to their various institutions. Students also expressed satisfaction on the potential immediate and long-term impacts of the school. They confirmed that the experience was remarkable on several fronts. First, the school helped in sculpting and redirecting their focus as neuroscientists. Their biggest takeaway messages from the school were that ‘research goals can be achieved with commitment and consistency, ‘little beginnings should not be jettisoned’, and ‘being diligent with the little we are doing because it might be the missing link in another work elsewhere which could facilitate an unthinkable collaboration’. To this, they expressed optimism that ‘no matter the dearth in resources, a lot of research breakthroughs are still possible within Africa’.

Secondly, regarding the impact of the school on the immediate career, students were able to interact, share ideas on ongoing projects, available equipment, and initiate collaborations. It is noteworthy that insights were discussed with facilitators on applying the learnt skills, techniques and knowledge to ongoing or fresh projects and grant applications.

After the training, a WhatsApp group was created where all attendees can further share research opportunities, brainstorm on calls for research proposals especially by keying into the Tertiary Education Trust Fund (TETFund) yearly research grants/interventions of the Nigerian Federal Ministry of Education, and as a means of keeping track of trainees’ career progression, with Professor James Olopade serving as the group’ mentor.

Furthermore, on the participants feedback, they were of the view that establishment of centres of excellence across Africa focusing on NSC research by the African government or other funding agencies will foster research collaborations on a more sustainable basis, as this will provide an ideal environment attracting NSC researchers from outside Africa as postdoctoral fellows or visiting scientists for cross-fertilization of ideas. Likewise, more graduate schools with a focus on neuroscience and NSC research are needed in order to train more masters students and PhD candidates at African universities. The immense demand for well-trained NSC researchers can only be met by increasing the output of masters and PhD graduates at African universities. Equally, quality undergraduate education necessitates a curriculum that embeds preliminary NSC knowledge enriched by research findings. This would require lecturers with an NSC research background. International collaboration is a prerequisite for effective capacity building, therefore, there is a need to firmly establish graduate schools in the global neuroscience setting (Rüland, 2013). The establishment of postdoctoral and other research opportunities, including research visits to foreign laboratories to build up new networks, could partially address this backwardness. Encouraging and enabling the returning fellows to implement and propagate NSC research would have a positive impact. While recognizing the demand of teaching responsibilities, efforts should be made to encourage and strengthen innovative research among these early-career researchers through proper mentoring.

### Recommendations and concluding remarks

Globally, stem cells have revolutionized biomedical research, as the past 20 years have witnessed astounding innovations in pursuit of stem cell applications in both health and livestock production. Also, mesenchymal stem cells (as a result of their attainment ease and pluripotency) have emerged as clinically important cells for companion animals’ and pets’ injury treatments.

Overall, we have ascertained that basic experimental studies using the NSCs approach are not yet established on the African continent.

In conclusion, we recommend the following:

1. Other African countries can take a cue from South Africa and Nigeria in probing the phenomena of adult neurogenesis in unique animal species on the continent, as a research topic of interest.
2. Leveraging the expertise and facilities of international collaborators in scaling up NSC research into these unique species.
3. Setting up of Centers of Excellence on the continent that can serve as research hubs to train postgraduate students and be retraining centers for those who have gone abroad and trained in NSCs but returned to the African continent.
